# Can a single question effectively screen for burnout in Australian cancer care workers?

**DOI:** 10.1186/1472-6963-10-341

**Published:** 2010-12-16

**Authors:** Vibeke Hansen, Afaf Girgis

**Affiliations:** 1 Centre for Health Research & Psycho-oncology(CHeRP), The Cancer Council NSW, University of Newcastle & Hunter Medical Research Institute, David Maddison Building, Callaghan NSW 2308, Australia

## Abstract

**Background:**

Burnout has important clinical and professional implications among health care workers, with high levels of burnout documented in oncology staff. The aim of this study was to ascertain how well a brief single-item measure could be used to screen for burnout in the Australian oncology workforce.

**Methods:**

During 2007, 1322 members of the Clinical Oncological Society of Australia were invited to participate in a cross-sectional nationwide survey; 740 (56%) of eligible members consented and completed the survey. Data from the 638 consenting members who reported that their work involved direct patient contact were included in the secondary analyses reported in this paper. Burnout was assessed using the MBI Human Services Survey Emotional Exhaustion sub-scale and a single-item self-defined burnout scale.

**Results:**

Emotional exhaustion was "high" in 33% of the sample when assessed by the psychometrically validated MBI. The single-item burnout measure identified 28% of the sample who classified themselves as "definitely burning out", "having persistent symptoms of burnout", or "completely burned out". MBI Emotional Exhaustion was significantly correlated with the single-item burnout measure (r = 0.68, p < 0.0001) and an ANOVA yielded an R^2 ^of 0.5 (p < 0.0001).

**Conclusions:**

The moderate to high correlation between the single-item self-defined burnout measure and the emotional exhaustion component of burnout suggest that this single item can effectively screen for burnout in health care settings which are time-poor for assessing burnout more comprehensively.

## Background

Professional burnout is an important issue in health and human service professions, due to its strong association with outcomes such as increased medical errors [1], decreased quality of patient care [2] and increased turnover and absenteeism [3]. The high prevalence of burnout in cancer care workers is a particularly salient issue due to the highly vulnerable patient groups involved and the importance of retaining skilled and experienced personnel in an occupational field marked by existing staff shortages.

International research has clearly established high levels of burnout and psychological distress in oncology staff [4-6]. More than half (56%) of oncologists in a US sample reported experiencing an episode of burnout at some stage during their career, with incidence of burnout rising with increasing time spent in direct patient contact [7]. Similarly, high levels of occupational distress are reported by Australian cancer care workers, with 33% of those whose work involves direct patient contact and 27% of those without patient contact exhibiting high levels of emotional exhaustion [8].

Historically, professional burnout in health care providers has been assessed via the Maslach Burnout Inventory - Human Services Survey (MBI-HSS), which is a well-validated 22-item self-report questionnaire with strong psychometric properties [9]. Its three sub-scales measure distinct, but interrelated, aspects of burnout: emotional exhaustion (EE), depersonalisation, and personal accomplishment. However, with a completion time of 10-15 minutes, the MBI-HSS does not easily lend itself to routine administration for screening purposes in most health care settings, where time allocated to staff welfare and occupational health and safety often is at a premium.

Of the three MBI sub-scales, EE has been widely regarded as the core component of the multidimensional construct of burnout [10,11], with fatigue and exhaustion reported as central features of burnout [12]. The important role of EE is also evidenced by previous findings that self-diagnosis of burnout is based on EE [13,14]; and only the EE component of burnout was predictive of cancer care workers' intention to leave the profession in a Canadian study [4].

Previous research indicates that the single-item measure of self-defined burnout developed initially for the Physician Worklife Study [15] may be a satisfactory screening tool for burnout, due to its demonstrated positive association with the EE sub-scale of the MBI-HSS in a US sample of physicians [14]. However, the potential usefulness of this brief screening measure being administered routinely in Australian clinical settings is unknown, given lack of Australian data on its validity as a burnout measure.

This paper reports on the predictive validity of self-defined burnout assessed using a single item by measuring its association with the EE sub-scale of the Maslach Burnout Inventory in a sample of Australian cancer care workers.

## Methods

### Sample & Procedure

The Clinical Oncological Society of Australia (COSA) is the peak national body representing health professionals from a range of multidisciplinary groups, whose main work is in the area of cancer control. COSA members (N = 1,322 at May 2007) received a letter from the COSA secretariat with initial information about the study. Contact details of members who had not declined further contact (n = 1,157) were sent to the researchers for all further communication regarding the study. Members received study information (n = 1,059 by email; n = 98 by post), including a URL for accessing the web-based survey and a personal log-in and password. Non-responders received reminders two, three and six weeks after the initial invitation date. Completion of the survey was taken as consent to participate. No further contact was made with non-responders. The University of Newcastle Human Research Ethics Committee approved the study.

### Instruments

#### Maslach Burnout Inventory

The 22-item Human Services version (MBI-HSS) [9] was administered to respondents who reported having direct patient contact as part of their work. The MBI-HSS consists of three sub-scales: Emotional Exhaustion, Depersonalisation, and Personal Accomplishment. The cut-off scores recommended by the MBI scale developers were applied to indicate low, average or high levels of burnout on each sub-scale separately [9]. The sub-scales were scored as recommended by the developers of the scale; and only the EE sub-scale data are reported in this paper.

#### Self-defined burnout

A single item developed by Schmoldt et al [15], was included to assess self-defined burnout, with five response options: (i) I enjoy my work. I have no symptoms of burnout; (ii) Occasionally, I am under stress, and I don't always have as much energy as I once did, but I don't feel burned out; (iii) I am definitely burning out and have one or more symptoms of burnout, such as physical and emotional exhaustion; (iv) The symptoms of burnout that I'm experiencing won't go away. I think about frustration at work a lot; (v) I feel completely burned out and often wonder if I can go on. I am at the point where I may need some changes or may need to seek some sort of help.

#### Statistical analyses

Analyses were conducted using SPSS software. Mean score and prevalence of high burnout on the MBI-EE were calculated. An ANOVA analysis compared the MBI-EE sub-scale to the categorical responses to the self-defined burnout item. ANOVA was considered appropriate, as the data displayed normally distributed residuals and homogeneity of variance. A Pearson correlation coefficient was also calculated as a measure of association. A false negative score on the self-defined burnout item was defined as a score of 1 on the single item and either average or high on EE, or 2 on the single item and high on EE. A false positive score was defined as a score of 5 on the single item and either low or average on EE, or 4 on the single item and low on EE.

## Results

### Sample

Of the 1157/1322 financial COSA members willing to receive the initial survey invitation, 9 were ineligible and 165 declined contact from the research team. A total of 740 surveys were completed, representing a response rate of 56% of the known eligible COSA membership and a consent rate of 64.5% of eligible members who received the study information. The secondary analyses reported here are of the sub-sample of 638 participants who reported that their work involved direct patient contact. Participant demographic and occupational characteristics are in Table 1.

**Table 1:** Demographic and occupational characteristics of eligible respondents (n = 638)

Characteristic	N	%
Gender: Female	499	78
Occupational group		
Nurse	361	56.6
Oncologist and palliative care physician	147	23.0
Other health professionals	78	12.2
Research and administration	45	7.1
Other	7	1.1
Location		
Rural/remote	126	19.7
Metropolitan	512	80.3
		
	**Mean**	**SD**
Age	45.7	9.8
Years in current occupation	13.6	10.5
Years in cancer care	14.2	8.2

### Burnout - EE versus single item

The EE mean score of 21.3 (SD = 11.7) in this Australian sample is similar to that from published norms (Mean = 22.19, SD = 9.53, *p = 0.07*) from 1104 physicians and nurses in the USA [9]. Almost one-third of our sample (32%, n = 204) was identified as having high levels of burnout on the EE sub-scale, compared to 28.2% (n = 180) classifying themselves as definitely burning out (20.7%), having persistent symptoms of burnout (4.7%), or being completely burned out (2.8%) on the self-defined burnout item (Mean = 2.3, SD = 0.8).

As shown in Table 2, results of the ANOVA procedures comparing self-defined burnout and EE sub-scale scores indicate that the Emotional Exhaustion sub-scale of the MBI and the self-defined burnout measure indeed tap into a similar construct, with R^2 ^= 0.5 (p < .0001). This association is further evidenced by a significant moderate to high positive correlation between the two measures (r = 0.68, p < .0001).

**Table 2:** Mean MBI-EE sub-scale scores across the five self-defined burnout response categories (n = 622)*

MBI	Enjoy work n = 45	Some stress, not burned out n = 405	Definitely burning out n = 126	Burnout symptoms won't go away n = 28	Completely burned out n = 18	R^2^	r
	Mean (SD)	Mean (SD)	Mean (SD)	Mean (SD)	Mean (SD)		
Emotional Exhaustion	9.0 (6.7)	17.5 (8.8)	31.1 (8.0)	39.9 (6.4)	41.2 (8.2)	0.49**	0.68**

* 622 respondents completed both measures.

** Significant at p < .0001

In order to further assess how well the self-defined burnout measure performed against the EE sub-scale, the proportion of false negatives and positives detected by the self-defined burnout measure was calculated. The self-defined burnout measure detected one false positive, and 76 (12%) false negatives. The majority of these false negatives (11%) were respondents who had high MBI EE scores but defined themselves as only *occasionally under stress and sometimes lacking energy, but not feeling burned out*. This finding highlights the fact that fatigue and exhaustion are the central features underpinning the EE sub-scale, but suggests that some respondents indeed may experience "emotional exhaustion" without feeling burned out.

## Discussion

The significant positive correlation between self-defined burnout assessed by a single item and the emotional exhaustion component of burnout in this sample of Australian clinical cancer workers is consistent with previous US findings [14]. The finding of a minor proportion of false negatives on the self-defined burnout measure relative to the emotional exhaustion sub-scale appears to be an artifact of the inclusion of concepts relating to stress and fatigue in one of the low-burnout response options of the single-item measure. Further evaluation of this measure should therefore ideally incorporate assessment of the items' construct validity through inclusion of related objective measures of burnout, such as absenteeism.

Given the brevity and ease of administration of this measure, it has significant potential to be routinely used to effectively screen for burnout in health care settings which are time-poor for detecting symptoms related to fatigue and emotional exhaustion more comprehensively. Caution should be exercised in drawing inferences about the usefulness of the single item burnout measure in other health care settings, due to some under-represented professional groups in the current sample and the inherent self-selection bias introduced by the survey methodology and membership of COSA. However, the finding that the current sample reports levels of emotional exhaustion comparable to those in a large US normative sample of health professionals lend support to the validity of these findings across other health care settings.

## Conclusions

The current study indicates that a single-item measure of self-defined burnout can be effectively used to screen for burnout in an Australian oncology setting. Given the importance of preventing and addressing burnout in oncology settings, this easily administered burnout measure has got potential for being routinely administered as part of staff health and welfare procedures.

## Competing interests

The authors declare that they have no competing interests.

## Authors' contributions

Both VH and AG were involved in the conception and design of the study, and development of study measures. VH was responsible for data collection and analysis, and for drafting the manuscript. AG participated in data collection and analysis, and critically revised the manuscript. Both authors approved the final manuscript.

## Pre-publication history

The pre-publication history for this paper can be accessed here:

http://www.biomedcentral.com/1472-6963/10/341/prepub
